# The Language-Specificity of Phonetic Adaptation to Talkers

**DOI:** 10.1177/00238309231214244

**Published:** 2023-12-06

**Authors:** Anne Cutler, L. Ann Burchfield, Mark Antoniou

**Affiliations:** The MARCS Institute for Brain, Behaviour and Development, Western Sydney University, Australia

**Keywords:** Perceptual learning, first- and second-language listening, English, Mandarin

## Abstract

Listeners adapt efficiently to new talkers by using lexical knowledge to resolve perceptual uncertainty. This adaptation has been widely observed, both in first (L1) and in second languages (L2). Here, adaptation was tested in both the L1 and L2 of speakers of Mandarin and English, two very dissimilar languages. A sound midway between /f/ and /s/ replacing either /f/ or /s/ in Mandarin words presented for lexical decision (e.g., *bu4fa3* “illegal”; *kuan1song1* “loose”) prompted the expected adaptation; it induced an expanded /f/ category in phoneme categorization when it had replaced /f/, but an expanded /s/ category when it had replaced /s/. Both L1 listeners and English-native listeners with L2 Mandarin showed this effect. In English, however (with e.g., *traffic; insane*), we observed adaptation in L1 but not in L2; Mandarin-native listeners, despite scoring highly in the English lexical decision training, did not adapt their category boundaries for /f/ and /s/. Whether the ambiguous sound appeared syllable-initially (as in Mandarin phonology) versus word-finally (providing more word identity information) made no difference. Perceptual learning for talker adaptation is language-specific in that successful lexically guided adaptation in one language does not guarantee adaptation in other known languages; the enabling conditions for adaptation may be multiple and diverse.

## 1 Introduction

Everyday speech is enormously variable. Talkers differ in age, in sex, in the size and shape of their vocal tract, and in their current health and mood; all of these affect what their voice sounds like. They may use a dialect different to that of the listener, or they may have a foreign accent. Furthermore, the situation they are in as they talk may cause them to raise or lower their voice, or may influence their articulatory precision. All these factors affect how easily their speech is perceived by a given listener (Bradlow & Bent, 2008; Clopper et al., 2010). In short, every part of a spoken utterance may be realized in many different ways, and it is an everyday task for listeners to somehow accommodate to this variation, by mapping it to their already established representations of the categories of speech.

Speech recognition is nonetheless famously robust against variability. In most instances, coping with new talkers seems quite unproblematic and human listeners adapt rapidly to talkers they have never before encountered. Many lines of speech research have addressed how this adaptation is achieved. One such body of research concerns the deployment of lexical knowledge to resolve an ambiguity at the phonemic level, that is, at the level expressing a minimal difference between two otherwise identical spoken-word forms. This process is known as lexically guided perceptual learning.

This learning can be induced in the laboratory, in a simple two-part training/test paradigm developed by Norris et al. (2003). During part one, listeners are exposed to a speech sound that is ambiguous (e.g., between /s/ and /f/), occurring in a context that favors one interpretation over the other. For instance, L1 English listeners would interpret such an ambiguous sound as /f/ if it occurred at the end of *gira*-, because *giraffe* is an English word but *girasse* is not. After *hor*-, it would instead be interpreted as /s/ because *horse* is a word but *horf* is not. The initial training can involve simple listening or some task such as lexical decision.

In the following second part of the paradigm, the participants are tested on their perception of the ambiguous sound. This can happen, for instance, in a phoneme categorization test with a continuum (e.g., from a good /f/ at one endpoint to a good /s/ at the other). In that case, listeners will typically classify more medial continuum steps as the phoneme that was replaced by the ambiguous sound in the exposure material they heard. Differing categorization curves will then be observed across groups: those who heard the ambiguous sound replacing /f/ will reveal an enlarged /f/ category, while those who heard it as /s/ will have a larger /s/ category. Thus the boundary between two of the listeners’ phonemic categories (here, /f/ and /s/) has effectively shifted in opposite directions, respectively, expanding and shrinking, to accommodate the unusual talker-specific pronunciation heard in exposure. (See Clarke-Davidson et al., 2008 for tests of the nature of this shift.)

Perceptual learning does not depend upon particular listening strategies (Drouin & Theodore, 2018), so it can be induced with many different exposure types (Eisner & McQueen, 2006) and is captured by a variety of test tasks as well (McQueen et al., 2006; Sjerps & McQueen, 2010). The learning is stable over some time, largely speaker-specific, appears across the lifespan, and generalizes across the vocabulary; it has now been observed in a number of languages, and not only with phonemes but also with other types of speech sound, including tones (Mitterer et al., 2010), and also with written representations of speech sounds (Norris et al., 2006; on all these points see Cutler et al., 2010 for a review). Furthermore, this type of perceptual learning being just one instantiation of the ability of humans to use existing knowledge to adjust perceptual processing of any kind, it operates where possible in parallel with other types of adaptation (Ullas, Formisano, et al., 2020) with the different adaptations evoking the same cortical responses (Ullas, Hausfeld, et al., 2020).

Despite the robustness and generality of this learning about phonemes, it is also informative to note when it does not occur. Adaptation of front vowels does not alter perception of back vowels (Maye et al., 2010). Learning from ambiguous sounds does not occur when listeners have heard the same speaker produce good instances of the critical sound (Kraljic & Samuel, 2005). The adaptation also does not occur when the ambiguous pronunciation in exposure can be attributed to a transient cause (e.g., the speaker biting on a pen; Kraljic et al., 2008). Thus, the decision process underlying adaptation is sensitive to the larger perceptual picture, honing its efficiency.

Although perceptual learning for talker adaptation has been studied extensively for listeners in their L1, its usefulness would be at least as great in an L2, if not greater given the persistent disadvantages of L2 listening at the phonetic level (Best & Tyler, 2007; Garcia Lecumberri et al., 2010) and the lesser ability so often shown in recognizing talkers in L2 versus L1 input (Goggin et al., 1991). However, the extent to which lexically guided perceptual learning is applied in L2—that is, the relative flexibility of L2 listening—is as yet not well understood, not many studies having tackled this question to date. Does lexically guided perceptual learning occur as freely in L2 as in L1?

A first demonstration that L2 perceptual learning may occur was provided by Reinisch et al. (2013), testing German students in the Netherlands, thus immersed in their L2 Dutch (*M* immersion = 3.3 years). They showed perceptual learning with a Dutch /f/-/s/ contrast, and the L2 learning effect was commensurate with the same learning observed in Dutch L1 listeners with the same materials. Here the learning was thus found to occur in a homogeneous group of high-proficiency listeners, in a language environment presenting an immersion situation, and in two languages that are closely related.

Are these factors critical to the appearance of perceptual learning in L2? It seems not. Two further studies, albeit involving closely related languages from the West Germanic family, that is, some combination of Dutch, German, and English, have shown immersion situations not to be a necessary criterion. Schuhmann (2014) examined German listeners living in Germany, and Drozdova et al. (2016) tested Dutch listeners in the Netherlands, in both cases on an L2 English phonetic category contrast. Schuhmann’s listeners showed learning for English /f/-/s/, and Drozdova et al.’s for /l/-/ɹ/, despite the fact that neither listener group was immersed in an English-speaking environment. (In Drozdova et al.’s study, interestingly, the learning was asymmetrical, with one category more likely to expand than the other. The latter results possibly point to some limitation on the adaptation flexibility in L2.)

Not only the immersion factor but also overall linguistic family relations are unlikely to play an essential role; Chan et al. (2020) tested Cantonese-native but English-dominant residents in Vancouver, Canada, on perceptual learning for /f/, a phoneme that is highly similar in its Cantonese and English realizations despite the lack of relatedness of the two languages. Participants in their study succeeded in learning a deviant /f/ realization when exposed to it in words of Cantonese (nominally their L1 in terms of order of acquisition, but in fact neither their dominant language nor the language of their environmental immersion).

Thus perceptual learning might appear to be as robust as one could possibly want. It is rapid, it generalizes, it is long-lasting, it is available from childhood to older age, and listeners can make use of it in L1 or L2, in whatever environmental situation holds, and irrespective of language typology. The case for “language-specificity” in perceptual learning, as proclaimed in our title, may not seem hopeful. But note that none of the studies so far described have actually addressed the specificity issue in a direct manner. Most of those studies involved tests in only one language; where two languages were involved, it was the listener group that varied and not the test language, with the same language being examined in separate populations of (L1, L2) listeners.

Ideally, perceptual learning in L1 and L2 should be compared in the same listeners, for instance, to control for the individual differences that have been observed in the process of learning about individual talkers (Nygaard & Pisoni, 1998). Just one study has done that. Bruggeman and Cutler (2020) compared Dutch emigrants who had lived in Australia for an extended period of time. In fact, the mean length of residence was so long (*M* = 19.4 years) that the majority of the group (in which age varied from 24 to 73 years) had become L2-dominant, which has known effects on both speech perception and production of the L1 and L2 (Antoniou et al., 2010, 2012). These L2-dominant listeners indeed showed perceptual learning in their L2, English. Strikingly, however, they showed no such learning in their L1, Dutch.

Obviously, there are some differences between that participant group and those providing data for most of the rest of the perceptual learning literature. The average age of those emigrant participants was higher; and as emigrants they had left the land of their L1 and committed to a permanent life in the land of their L2. The language-specificity they evinced (of the starkest kind: yes for one language, no for the other) might be traceable to one or other characteristic of this group only. Bruggeman (2016) found no age-related effects within this set of learning data, nor any effects of hearing ability or of relative proficiency in either language (which was high in both for all listeners). It is an open question whether such language-particular adaptation patterns would similarly appear in other bilingual user populations, including younger high-proficiency users.

In the present study we address this issue. With younger populations who regularly use two languages, we test for what is implied by language-specificity in the case of perceptual adaptation to talkers using lexical information: Is perceptual learning applied in each of their known languages, to the same extent, and in the same way?

For this study we chose an unrelated language pair: English and Mandarin Chinese. These languages are from typologically quite separate linguistic families, and differ in very many respects, including phoneme repertoire size (English has a much larger repertoire of both vowels and consonants), syllable structure (English allows complex onsets and codas, Mandarin does not), morphology (English has both prefixes and suffixes, Mandarin has neither), as well as in the presence of lexical tone contrasts (lexically distinctive in Mandarin, absent from English). As in the standard procedure, we tested both ends of a phonetic continuum, training separate groups to hear an ambiguous sound as, respectively, /s/ or /f/. This already well-examined contrast exists in both the test languages. Within-repertoire competition for these fricatives is rather better balanced in the present language pair than it is in Bruggeman and Cutler’s Dutch versus English pairing; Mandarin has less competition with /f/ than English does, but, due to the number of sibilants in its phoneme inventory it has more competition with /s/ than English does (Maddieson, 1984). Such within-repertoire competition is an important factor affecting the manner of phoneme identification (Wagner, 2013), and in English and Dutch it is not well balanced, in that English has the sound /θ/, and the /θ/-/f/ contrast is the most confusable in the language, whereas Dutch has no close competitors for either /f/ or /s/.

Perceptual learning has been observed in L1 Mandarin for ambiguous tone contrasts in the same way as for the phonetic contrasts described so far (Mitterer et al., 2010), and perceptual learning for Australian English, the variety used here, has also been established (McQueen et al., 2012). Each of the present experiments followed Bruggeman and Cutler’s (2020) design by examining lexically guided perceptual learning within the same groups of users in both their languages, except of course that the languages here were typologically unrelated and phonologically quite different.

Both listener groups lived in their L1 environment, that is, neither was in an immersion situation. In Experiment 1, participants were Australian English speakers resident in Sydney, who were learning or had learned Mandarin as an L2. In Experiments 2 and 3, our participants were university students in Hong Kong. Although Hong Kong is a culturally diverse city with a sizable English expatriate population, the majority of residents are native Chinese (Cantonese and/or Mandarin speakers); our listeners all had Mandarin as L1.

## 2 Experiments

Perceptual learning experiments require a standard against which to measure the effect of the induced learning. This is mostly a pretest with the phonetic categorization task, enabling selection of an ambiguous phoneme [?] that is accepted as halfway between the endpoints of the phonetic continuum to be manipulated; the pretest is carried out either with a separate group representative of the listener population (Norris et al., 2003), or individually for each participant (Bruggeman & Cutler, 2020; Ullas, Formisano, et al., 2020). Bruggeman and Cutler showed that the choice of pretest method does not influence results; here we used separate pretest groups for each language.

There are also several options for the choice of exposure to induce learning. The most widely used task has been lexical decision, but given that lexical decisions in L2 are typically made more slowly and less confidently than in L1 (Harrington, 2006), we also considered other methods, such as listening to a story (Eisner & McQueen, 2006, e.g., used *The Little Prince*). However, these too present problems of equivalence across languages (e.g., not being equally familiar to audiences with differing cultural backgrounds). Drozdova et al. (2016) used a story as exposure material in their L1–L2 comparison, but they tested in only one language and thus had only one version of the story. Construction and calibration of matching stories across English and Mandarin (with their different phonological word position constraints, for instance) would be a formidable task both phonologically and sociolinguistically. Reinisch et al. (2013), Schuhmann (2014), Bruggeman and Cutler (2020), and Chan et al. (2020) having all successfully reported perceptual learning from a lexical decision task exposure in a L2/non-dominant language, we chose to use that procedure also.

### 2.1 Pretests

Our ambiguous phoneme [?] was to be halfway between [f] and [s], and our material sets were Mandarin and English words. Fricative contrasts in Mandarin can only appear syllable-initially; the fricatives in the English words therefore also occurred only in syllable-initial position. We carried out a separate /f/-/s/ categorization pretest for each materials set.

#### 2.1.1 Participants

Participants in the pretest experiments were 16 native speakers of Australian English located in Sydney (10 females, 6 males, *M* age = 28.9 years, age range 18–46) and 16 native Mandarin speakers located in Hong Kong (11 females, 5 males, *M* age = 25.6 years, age range 24–32); none reported any known vision, language, or hearing impairments.

#### 2.1.2 Materials

For each language, a female native speaker produced the syllables /fu/, /su/, and /θu. All tokens were natural utterances and were recorded in a sound-attenuated room at a sampling rate of 44.1 kHz. The fricative portions (starting at a rising zero-crossing at the onset of frication and ending at the offset of frication) were extracted from tokens of /fu/ and /su/ (for each language) using Praat (Boersma & Weenink, 2001).

From each [f]-[s] pair, a 41-step continuum was created following the procedure of Norris et al. (2003). The [f] and [s] waveforms were mixed at proportions equally spaced along a 41-step continuum such that one end token was 100% [f] and the other was 100% [s]. Each fricative token was scaled down to 35% of its original amplitude and concatenated with a token of [u] extracted from the production of /θu/ (for each language). This avoided coarticulatory cues in the vowel that might bias listeners’ interpretation toward [f] or [s].

#### 2.1.3 Procedure

Listeners heard their L1 continuum only. Tokens were presented at a comfortable listening level through headphones at the rate of one every 2.6 seconds. Participants were asked to identify each auditory token as either /fu/ or /su/ by pressing the “F” or “S” keys, as quickly and accurately as possible. No feedback was provided. For each continuum, there were 10 blocks of the 14 continuum steps, giving 140 trials in total. The order of presentation of the tokens within each block was randomized. The stimuli were presented using E-Prime.

#### 2.1.4 Results

**Figure 1.**
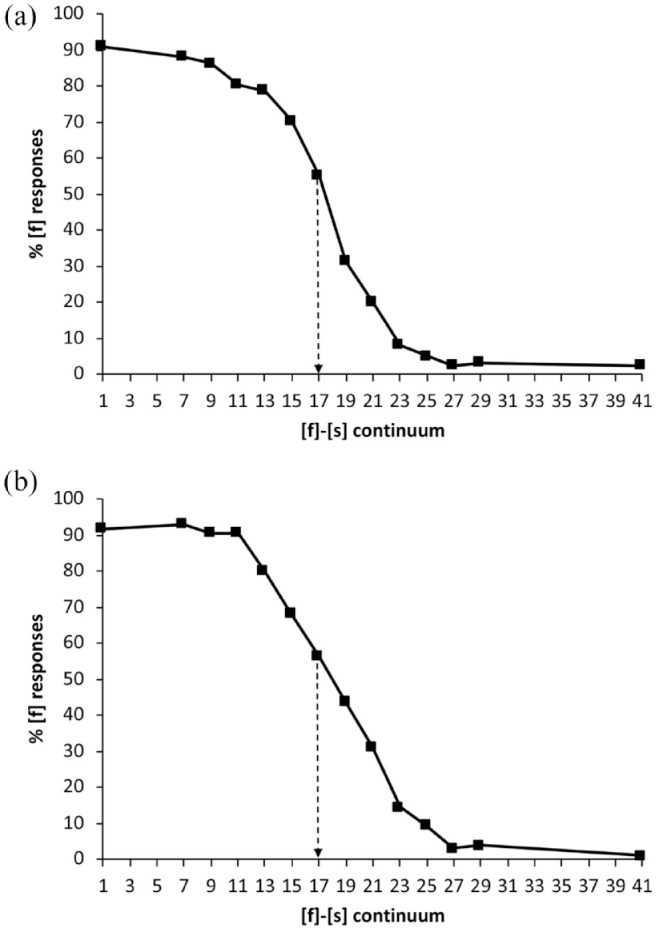
Total proportions of [f] responses, averaged across participants for the pretested continua in (a) Mandarin and (b) Australian English. The point indicated by the dotted arrow was selected in each case as the ambiguous fricative [?] for use in the training stimuli.

Figure 1 shows the /f/ response proportions from the 14 pretest continuum steps, averaged across each group. For each continuum, Step 17 proved to be closest to 50% [f], and hence 50% [s], responses. Each Step 17 was thus judged the most ambiguous token and was used as [?] in training and post-tests. Steps closest to 0%, 25%, 75%, and 100% were also identified.

## 3 Perceptual learning Experiment 1: English L1, Mandarin L2

### 3.1 Method

#### 3.1.1 Participants

In all, 30 native Australian English speakers born in Australia were recruited from the community using recruitment flyers (6 were university students) to take part in this experiment (17 females, 13 males, *M* age = 29.1 years, age range 19–50). All reported English as their dominant language and Mandarin as L2. The age at which they started to learn Mandarin varied widely (age range = 6–25) and in many cases gaps of several years separated periods of Mandarin instruction. Participants were asked to rate their proficiency in English and Mandarin on a scale from *very poor* to *excellent*; their mean self-ratings were always higher for English than for Mandarin. No participant reported any vision, language, or hearing impairments. One further recruited participant was excluded due to a data recording error.

#### 3.1.2 Materials

English training materials consisted of 100 real English words and 100 phonotactically legal nonwords. Critical items included 40 disyllabic words containing [f] (f-words, e.g., *infant*) or [s] (s-words, e.g., *basic*). Substituting [s] for [f] in the f-words, or vice versa, would produce a nonword. The two sets of 20 words were closely matched for frequency (f-words: 4.3 per million, s-words: 4.1). The remaining 60 real English words and 100 nonwords served as filler items and varied in length from one to four syllables. To avoid any sounds perceptually similar to the critical fricatives, filler items contained no [f], [s], [v], [z], [ʃ], [ʒ], [θ], or [ð].

Mandarin training materials were created following similar criteria. All words and nonwords were disyllabic. In critical items, [f] and [s] occurred word-medially (as the first phoneme of the second syllable, e.g., 场所 [chong3suo3],“place”; 处分 [chu3fen4], “punish”). Tone of the final syllable was balanced across f- and s-words. For each set, six words contained Tone 1, two Tone 2, five Tone 3, and seven Tone 4. The f- and s-words were again balanced in word frequency (3.68 per million for f-words, 3.82 per million for s-words). The 60 unmodified Mandarin words and 100 nonwords contained no [f], [s], [ʂ], [ɕ], [ts], or [ts^h^]. Appendix A contains a full list of stimuli in both languages.

#### 3.1.3 Procedure

The main experiment consisted of two sessions, spaced approximately 2 weeks apart, differing in which stimuli were presented. Session order was counterbalanced across participants: half completed the English session first, and half the Mandarin session. A language background questionnaire was completed during the first session. During each session, participants first carried out an auditory lexical decision task in which they were trained to interpret [?] as /f/ or as /s/. In the following test phase, a phonetic categorization task assessed their perceptual learning.

#### 3.1.4 Training phase: auditory lexical decision task

Four stimulus lists were constructed for the auditory lexical decision task. Each list contained the same 100 words and 100 nonwords. The items were arranged in two different pseudorandom orders, such that no more than four words or four nonwords were presented successively. For each of these two orders, two versions were created, one in which all 20 instances of [f] were replaced with [?] and one in which all 20 instances of [s] were replaced with [?]. The initial 12 trials, which included both words and nonwords, were the same across all four lists and did not contain either [f] or [s]. Half of the participants (the f-trained group) heard a list in which [f] had been replaced by [?], while the other half (the s-trained group) heard a list in which [s] was replaced by [?]. Both groups heard the same set of filler items and nonwords. Tokens were presented via headphones at the rate of one every 2.6 seconds.

Participants were tested in a quiet room and were instructed that they would be presented with auditory words or nonwords. They were asked to indicate whether each heard item was an existing real word by pressing one of two buttons labeled *Yes* or *No* (in the relevant language) on a button box as quickly and as accurately as possible. *Yes* responses were made with the dominant hand. No feedback was given to participants and they were not made aware of the presence of the ambiguous fricative.

#### 3.1.5 Test phase: phonetic categorization task

Following the training, participants completed a 150-trial phonetic categorization task, in which Steps 7, 13, 17, 21, and 27 of the relevant continuum were presented 30 times each, via headphones, again in random order at the rate of one every 2.6 seconds. Participants were asked to categorize each consonant sound as [f] or [s] by pressing the “F” or “S” key.

### 3.2 Results

#### 3.2.1 Lexical decision task

Mandarin lexical decision performance across training conditions is summarized in Table 1.

**Table 1.** Percentage of Correct Responses and Response Times (RT), Measured from Target Word Onset, to Experimental Items in the Mandarin Lexical Decision Task.

Fixed effect	[f]-trained group		[s]-trained group	
	Natural fricatives	Ambiguous fricatives	Natural fricatives	Ambiguous fricatives
*M* % “yes”	52.9	52.6	53.2	41.8
*M* RT “yes” (ms)	1,619.6	1,567.3	1,455.1	1,609.0

We fitted a generalized linear mixed-effects (*glmer*) model with the logit-link function, implemented in the lme4 package (Bates et al., 2015) in R (R Core Team, 2018). Fixed factors included training condition ([f]-bias vs. [s]-bias; deviation coded: −0.5, 0.5) and pronunciation (natural vs. ambiguous; deviation coded: −0.5, 0.5). A maximal random effects structure was used (Barr et al., 2013), with random intercepts for participants and items, as well as random slopes for pronunciation by participant. Results of the model fit are displayed in Table 2. Although the lexical decision scores are not as high as what might be expected of native speakers, the patterns within the data suggest that participants judged approximately half of the instances of the ambiguous fricatives to be acceptable instances of Mandarin [f] or [s].

**Table 2.** Fixed-Effect Estimates of English-Mandarin Listeners’ Accuracy in the Mandarin Lexical Decision Task.

Fixed effect	β	*SE*	*z*	*p*
Intercept	−0.054	0.162	−0.330	.742
Training condition	0.126	0.292	0.433	.665
Pronunciation	0.276	0.161	1.711	.087
Training condition: Pronunciation	0.490	0.407	1.205	.228

*SE*: standard error.

Listeners’ responses in the English lexical decision task are displayed in Table 3. It is clear that these Australian learners of Mandarin show a very clear processing advantage for their native language, English. Their lexical decision performance is near ceiling for all stimulus categories except when the ambiguous fricative [?] replaced [s] (75.7%).

**Table 3.** Experiment 1: Percentage of Correct Responses and Response Times (RT), Measured from Target Word Onset, to Experimental Items in the English Lexical Decision Task.

Fixed effect	[f]-trained group		[s]-trained group	
	Natural fricatives	Ambiguous fricatives	Natural fricatives	Ambiguous fricatives
*M* % “yes”	96.7	98.0	98.3	75.7
*M* RT “yes” (ms)	1,157.8	1,183.6	1,079.5	1,265.7

We fitted a *glmer* model to the English lexical decision data also, using the same fixed factors and random structure as for the Mandarin analysis; Table 4 displays results of the model fit. The Australian learners of Mandarin judged the ambiguous fricative [?] as an acceptable instance of English [f] or [s]; the significant effects reflect that the percentage of “yes” responses were lower for the [s]-ambiguous items than for the other conditions.

**Table 4.** Fixed-Effect Estimates of English-Mandarin Listeners’ Accuracy in the English Lexical Decision Task.

Fixed effect	β	*SE*	*z*	*p*
Intercept	−4.087	0.402	−10.180	<.001
Training condition	1.426	0.550	2.591	.010
Pronunciation	1.311	0.628	2.087	.037
Training condition: Pronunciation	3.859	1.153	3.346	<.001

*SE*: standard error.

#### 3.2.2 Post-test

**Figure 2.**
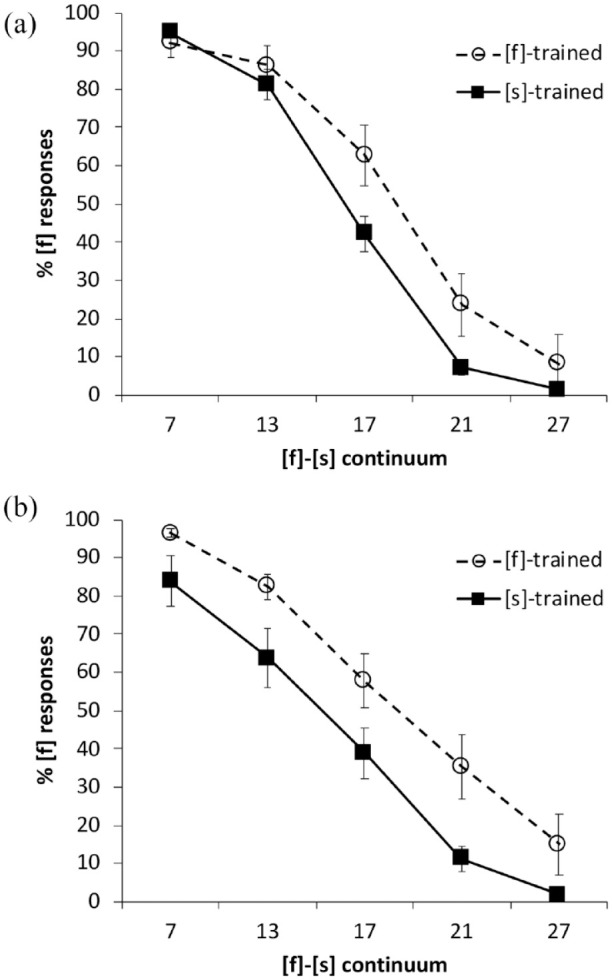
Percentage of [f] responses by the English-native listeners along the [f]-[s] continuum in: (a) Mandarin and (b) English. Error bars show standard error of the mean.

Figure 2 shows the categorization results for (A) Mandarin and (B) English. To compare categorization responses across languages, and between the [f]- and [s]-trained conditions, we fitted a *glmer* model with family “binomial” and the logit-link function. The following were included as deviation-coded fixed factors: continuum step, which was centered on Step 17 (Step 7 coded as −2, Step 13 as −1, Step 17 as 0, Step 21 as 1, and Step 27 as 2); training condition ([f]-trained coded as −0.5, [s]-trained as 0.5), and language (Mandarin coded as −0.5, English as 0.5). Random intercepts were added for participants and items, and random slopes for continuum step and language by participant, and for training condition by item.

Results of the model fit, shown in Table 5, revealed significant effects of continuum step and training condition, modulated by higher-order interactions of language and continuum step, training condition and continuum step, plus a three-way interaction. To explore lexically guided perceptual learning effects in each language, we next examined categorization in L1 and L2 separately using *glmer* models with the same parameters as above, but without the language factor; see Tables 6 and 7.

**Table 5.** Fixed-Effect Estimates of Listeners’ Categorization at Post-Test in Both Mandarin and English.

Fixed effect	β	*SE*	*z*	*p*
Intercept	0.013	0.288	0.046	.963
Continuum step	−1.896	0.124	−15.314	<.001
Training condition	1.134	0.435	2.605	.009
Language	−0.161	0.361	−0.445	.656
Continuum step: Training condition	0.246	0.109	2.253	.025
Continuum step: Language	0.370	0.075	4.944	<.001
Training condition: Language	0.690	0.879	0.786	.432
Continuum step: Training condition: Language	−0.655	0.197	−3.322	<.001

*SE*: standard error.

**Table 6.** Fixed-Effect Estimates of Categorization Scores by Australian Learners of Mandarin at Post-Test of the Five Steps Selected from the Mandarin [fu]-[su] Continuum.

Fixed effect	β	*SE*	*z*	*p*
Intercept	0.007	0.305	0.023	.981
Continuum step	−2.255	0.203	−11.127	<.001
Training condition	1.361	0.551	2.472	.013
Continuum step: Training condition	0.342	0.358	0.957	.338

*SE*: standard error.

**Table 7.** Fixed-Effect Estimates of Categorization Scores by Australian Learners of Mandarin at Post-Test of the Five Steps Selected from the English [fu]-[su] Continuum.

Fixed effect	β	*SE*	*z*	*p*
Intercept	−0.019	0.299	−0.065	.948
Continuum step	−1.670	0.135	−12.349	<.001
Training condition	1.321	0.572	2.311	.021
Continuum step: Training condition	−0.019	0.245	−0.079	.937

*SE*: standard error.

For the listeners’ L2, Mandarin, there was a significant effect of continuum step (Table 6), indicating that listeners were sensitive to progression across the Mandarin [f]-[s] continuum. There was also a significant effect of training condition, revealing that perceptual learning occurred in Mandarin (note the separation of the lines in the top panel of Figure 2).

Similarly, for the listeners’ L1, English, we observed significant effects of continuum step and training condition (see Table 7 and the bottom panel of Figure 2). For the Australian learners, that is, clear evidence of perceptual learning appears in both the L1 and the L2.

Scharenborg and Janse (2013), in their study of perceptual learning in older versus younger listeners, developed a measure of the degree of learning in the test phase (the mean training-consistent response, using the average of those responses made to the three most ambiguous test alternatives which were choices for the phoneme alternative consistent with training), and found it to be related to word acceptance rate in lexical decision training. Calculating this measure for the present data set, however, produced no significant correlation, *r*(28) = −.217, *p* = .26 for Mandarin, *r*(28) = .095, *p* = .62 for English.

### 3.3 Discussion

Australian English-native learners of Mandarin, presented with ambiguous fricatives in both Mandarin and English, showed lexically guided perceptual learning in both their L1 and L2, offering the first instance of such a pattern to be established. These results differ from those of Bruggeman and Cutler (2020) not only in the cross-language positive learning effect, but also in that the two languages involved are typologically distant, and the participants are young listeners, living in their L1 environment.

## 4 Perceptual learning Experiment 2: Mandarin L1, English L2

In Experiment 2, we tested listeners who were users of the same two languages, Mandarin and English, but with an order of acquisition that was the reverse of that in Experiment 1. Native speakers of Mandarin with English as their L2, again residing in an environment which did not immerse them in their L2, heard the same two sets of language materials as were used in Experiment 1.

### 4.1 Method

#### 4.1.1 Participants

In all, 28 native Mandarin listeners born in Mainland China and resident in Hong Kong (*M* age = 22.6 years, age range 19.9–28.2) took part in Experiment 2. None reported any language, vision, or hearing impairment. All were students at the Chinese University of Hong Kong (CUHK) and all were female. CUHK offers tuition in English and requires a minimal standard of English proficiency from all students (see Appendix B). All participants reported Mandarin as their dominant language and none reported experience with languages or dialects other than standard Mandarin before school age. Their self-reported mean English proficiency was 6.0 on a scale from 1 *very poor* to 9 *excellent*.

For this group we also collected data using the Versant speaking test, a commercially available automated test of spoken languages. The test may be administered either via telephone or by computer with a headset, and scores are calculated by the proprietary software and made available online. The Versant English Test automatically evaluates the spoken English skills of non-native English speakers, using speech processing technology. A score is generated indicating overall spoken language ability. Scores range from 20 to 80 (the higher the score, the greater the spoken language ability). Participants’ mean score on this test was 48.1 (*SD* = 9.3), which classifies them as intermediate users of English able to “handle many utterances using a variety of words and structures,” “follow and sometimes participate in a native-paced conversation,” with a pronunciation that is “mostly intelligible.”

Data of an additional 12 recruited participants were not used, due to experimenter error (two), misunderstanding of the task (four), another Chinese language (Cantonese, Hubei) as L1 (three), poor scores in the English lexical decision training task (one), and failure to return for second session (two).

#### 4.1.2 Materials and procedure

Materials and procedure were as in Experiment 1, with the exception that prior to the categorization task, participants also participated in a short cross-modal priming task based on that of Sjerps and McQueen (2010). That task is not reported in detail here, but the results paralleled those of the phonetic categorization task as reported below (see Burchfield et al., 2017).

### 4.2 Results

#### 4.2.1 Lexical decision task

Table 8 shows the Mandarin–English listeners’ responses to the Mandarin lexical decision items in the training task. As in Experiment 1, native speakers accepted nearly all instances of [f] and [s], including those containing the ambiguous phoneme [?], when hearing their L1.

**Table 8.** Percentage of Correct Responses and Response Times (RT), Measured from Target Word Onset, to Experimental Items in the Mandarin Lexical Decision Task.

Fixed effect	[f]-trained group		[s]-trained group	
	Natural fricatives	Ambiguous fricatives	Natural fricatives	Ambiguous fricatives
*M* % “yes”	92.7	91.4	91.4	86.4
*M* RT “yes” (ms)	1,239.3	1,309.4	1,239.1	1,280.6

Again, a *glmer* model was fitted to the lexical decision data with the same deviation-coded fixed factors (training condition, pronunciation) and random effects structure as in Experiment 1. Results of this model fit are recorded in Table 9. There were no significant effects other than the intercept. This pattern of results suggests that participants judged the ambiguous fricatives to be acceptable instances of Mandarin [f] or [s].

**Table 9.** Fixed-Effect Estimates of Mandarin-English Listeners’ Accuracy in the Mandarin Lexical Decision Task.

Fixed effect	β	*SE*	*z*	*p*
Intercept	−3.472	0.415	−8.360	<.001
Training condition	0.407	0.422	0.965	.334
Pronunciation	0.562	0.307	1.830	.067
Training condition: Pronunciation	0.641	1.079	0.595	.552

*SE*: standard error.

Table 10 shows listeners’ responses to the English lexical decision experimental items. Here too a *glmer* model was fitted to the data, in the same way as for the Mandarin lexical decision task; the results of this model fit are displayed in Table 11. These listeners’ lexical decision performance in their L2 clearly outstripped that of the English-native participants for L2 Mandarin in Experiment 1.

**Table 10.** Percentage of Correct Responses and Response Times (RT), Measured from Target Word Onset, to Experimental Items in the English Lexical Decision Task.

Fixed effect	[f]-trained group		[s]-trained group	
	Natural fricatives	Ambiguous fricatives	Natural fricatives	Ambiguous fricatives
*M* % “yes”	82.9	83.3	88.2	70.0
*M* RT “yes” (ms)	1,285.0	1,288.3	1,168.3	1,388.8

**Table 11.** Fixed-Effect Estimates of Mandarin-English Listeners’ Accuracy in the English Lexical Decision Task.

Fixed effect	β	*SE*	*z*	*p*
Intercept	−2.121	0.262	−8.099	<.001
Training condition	0.143	0.312	0.459	.647
Pronunciation	0.675	0.218	3.105	.002
Training condition: Pronunciation	2.266	0.798	2.841	.005

*SE*: standard error.

As Table 11 shows, a significant effect of pronunciation appeared in the lexical decision results for English, mediated by an interaction between training condition and pronunciation, the source of which was that words ending in naturally pronounced fricatives were accepted as existing words more often than words ending in ambiguous fricatives for the [s]-trained group only.

### 4.3 Post-test

**Figure 3.**
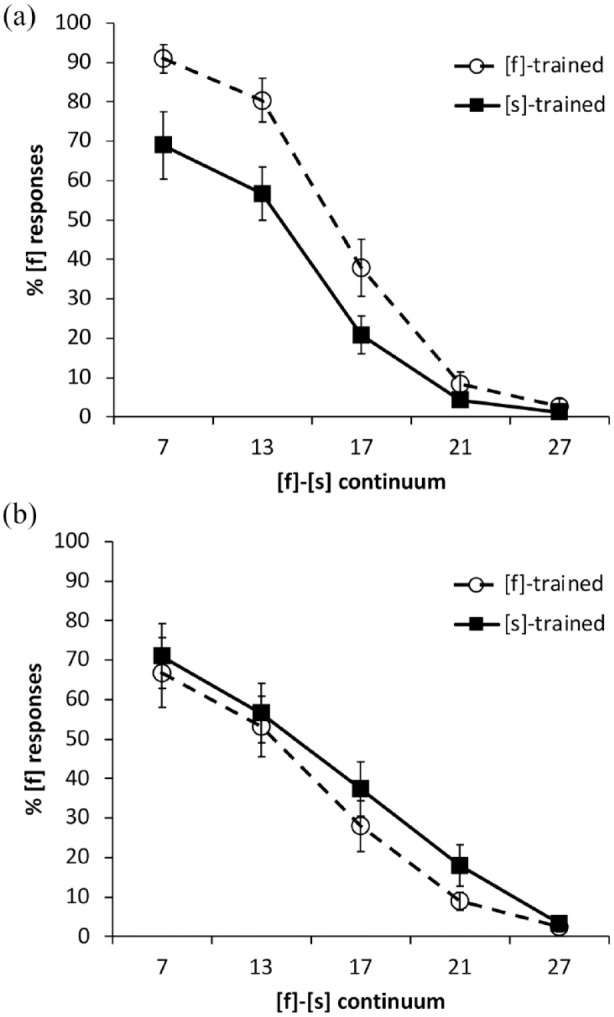
Experiment 2: Percentage of [f] responses for the two Mandarin-native training groups along the [f]-[s] continuum in: (a) Mandarin and (b) English. Error bars show standard error of the mean.

Results for the categorization tests are shown in the two panels of Figure 3.

We compared categorization responses between languages and conditions with a *glmer* model as for Experiment 1. The results of the model fit (see Table 12) showed significant effects of continuum step and significant interactions between language and continuum step as well as language and training condition, confirming a difference in the shape of the categorization curves and the robustness of the training effects between the L1 and L2.

**Table 12.** Fixed-Effect Estimates of Listeners’ Categorization at Post-Test in Both Mandarin and English.

Fixed effect	β	*SE*	*z*	*p*
Intercept	−1.287	0.242	−5.319	<.001
Continuum step	−1.632	0.149	−10.963	<.001
Training condition	0.202	0.381	0.529	.597
Language	−0.021	0.371	−0.056	.955
Continuum step: Training condition	−0.085	0.111	−0.763	.445
Continuum step: Language	0.483	0.147	3.287	.001
Training condition: Language	−1.671	0.781	−2.140	.032
Continuum step: Training condition: Language	0.256	0.186	1.379	.168

*SE*: standard error.

We next explored lexically guided perceptual learning effects in each language by creating separate models for L1 and L2 as done in Experiment 1. For the listeners’ L1, Mandarin, there was a significant effect of continuum step. Importantly, there was a significant effect of training condition as well (see Table 13), indicating that perceptual learning occurred in Mandarin (note the separation of the lines in the top panel of Figure 3). For the L2, English, the only significant effect was that of continuum step (see Table 14). There was no evidence of perceptual learning in English. Calculation of the relation of lexical decision results to overall training-consistent categorization responses produced the same null results as before: *r*(20) = −.013, *p* = .96 for Mandarin, *r*(20) = −.041, *p* = .86 for English.

**Table 13.** Fixed-Effect Estimates of Mandarin-English Listeners’ Categorization at Post-Test of the Five Steps Selected from the Mandarin [fu]-[su] Continuum.

Fixed effect	β	*SE*	*z*	*p*
Intercept	−1.458	0.301	−4.839	<.001
Continuum step	−2.135	0.232	−9.187	<.001
Training condition	1.285	0.519	2.474	.013
Continuum step: Training condition	−0.622	0.405	−1.534	.124

*SE*: standard error.

**Table 14.** Fixed-Effect Estimates of Mandarin-English Listeners’ Categorization at Post-Test of the Five Steps Selected from the English [fu]-[su] Continuum.

Fixed effect	β	*SE*	*z*	*p*
Intercept	−1.286	0.318	−4.051	<.001
Continuum step	−1.365	0.163	−8.383	<.001
Training condition	−0.468	0.581	−0.805	.421
Continuum step: Training condition	−0.145	0.271	−0.537	.591

*SE*: standard error.

### 4.4 Discussion

Mandarin listeners here heard ambiguous fricatives in both Mandarin and English; the pattern of results may be interpreted as indicating that lexically guided perceptual learning occurred in their L1, but not in their L2. This contrasts with previous reports of strong learning effects in L2 English by German and Dutch listeners (Drozdova et al., 2016; Schuhmann, 2014), as well as with the findings of Experiment 1 in which “L1” and “L2” were the other way round to here and the same materials elicited positive effects in both languages.

The Experiment 1 and 2 populations were both in non-immersion environments; that does not seem to be a relevant factor for this differing pattern. The lexical decision scores did not seem to relate in any causative way to the strength of perceptual learning either in L1 or L2, and in any case the L2 lexical decision scores of the population that did show positive learning in L2 were lower than those of the population that failed to show it. One possible factor could reflect, albeit indirectly, the phonological differences between the two languages. Because Mandarin allows fricatives only in syllable-initial position, we had tried to match this factor across materials sets, and therefore constructed new English lexical decision materials in which the crucial sounds occurred word-medially and syllable-initially (e.g., *prefer, basic*). In all earlier English experiments, the ambiguous sounds had occurred word-finally (e.g., *autograph, eyewitness*); in many cases the word became uniquely identifiable before the crucial sound occurred. It is therefore very likely that the lexical information provided was stronger in those earlier sets, and added strength would of course be particularly useful for L2 listeners. The high L2 lexical decision scores in Experiment 2 may make this seem unlikely; however, we were able to test this proposal easily without the need to create new English materials, given that those devised for the study of Bruggeman and Cutler (2020) were available to us.

## 5 Perceptual learning Experiment 3: Mandarin L1, English L2

### 5.1 Pretest: English fricatives in syllable-final position

Because of the phonological illegality of syllable-final fricatives in Mandarin, a phonetic categorization pretest with Mandarin listeners was conducted to select an English syllable-final ambiguous phoneme [?]. 10 native Mandarin speakers in Hong Kong completed this English pretest (7 females, 3 males *M* age = 25.3 years, age range 23–29), which was modeled on the pretest for Experiments 1 and 2; a female native speaker of Australian English produced /εf/, /εs/, and /εθ/, the fricative portions were extracted and a 41-step [f]-[s] continuum created, each continuum step concatenated with an [ε] token from /εθ/.

**Figure 4.**
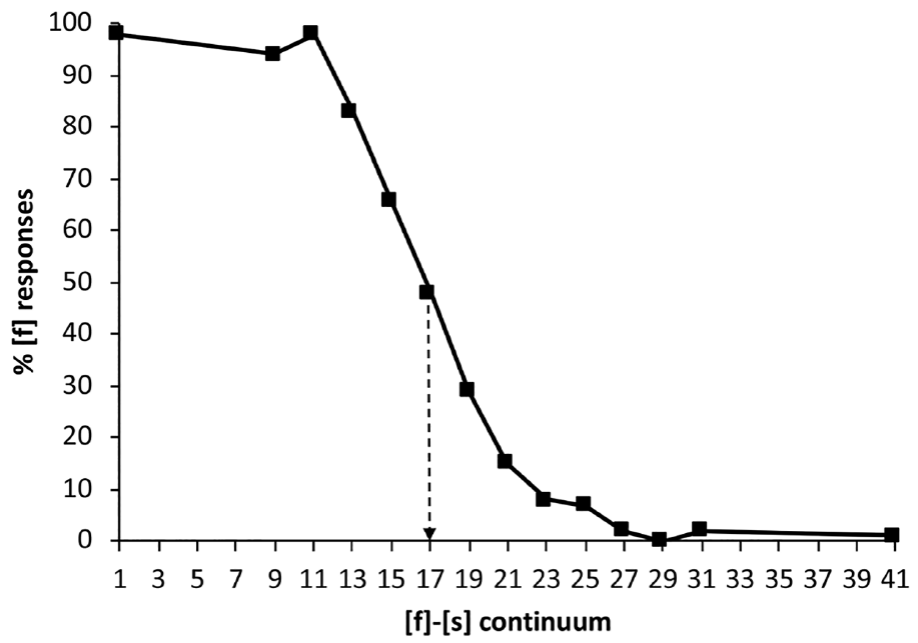
Total percentage of [f] responses, averaged across Mandarin participants for the English syllable-final continuum. Step 17 was selected as the ambiguous fricative [?]in the training stimuli.

Pretest participants were asked to identify each auditory token as /εf/ or /εs/ by pressing the “F” or “S” keys. As shown in Figure 4, Step 17 was deemed to be the most ambiguous token and was used as [?] in training and post-tests. Nonendpoint steps close to 0%, 25%, 75%, and 100% were also identified.

### 5.2 Method

#### 5.2.1 Participants

In all, 25 native Mandarin listeners born in Mainland China and residing in Hong Kong (22 females, 3 males *M* age = 24.3 years, age range 20–36) took part in Experiment 3. All were students at CUHK. All reported Mandarin as their dominant language and did not report use of languages or dialects other than standard Mandarin before school age. Their mean age of starting to learn English was 9.7 years (age range = 5–15). They rated their English proficiency on a scale from 1 *very poor* to 9 *excellent* at a mean of 5.2. None reported any vision, language, or hearing impairments. One further participant was excluded due to a technical issue.

#### 5.2.2 Materials, procedure and analyses

English materials resembled those of Experiments 1 and 2 except that the critical words ended in [f] (e.g., *autograph*) or [s] (e.g., *eyewitness*). The two sets of words were closely matched for frequency (4.3 per million for f-words, 4.1 for s-words). Mandarin materials were those used in Experiments 1 and 2. Experimental procedure was as in Experiment 1. The results were again analyzed with a *glmer* model with the same fixed effects and coding as before, and random slopes of participant by step were also included.

### 5.3 Results

#### 5.3.1 Lexical decision task

Table 15 shows listeners’ responses to the Mandarin lexical decision items containing the ambiguous sounds. We fitted a *glmer* model exactly as before. Results of the model fit are displayed in Table 16. There were no significant effects other than the intercept.

**Table 15.** Percentage of Correct Responses and Response Times (RT), Measured from Target Word Onset, to Experimental Items in the Mandarin Lexical Decision Task.

Fixed effect	[f]-trained group		[s]-trained group	
	Natural fricatives	Ambiguous fricatives	Natural fricatives	Ambiguous fricatives
*M* % “yes”	86.2	86.2	87.5	85.3
*M* RT “yes” (ms)	1,323.1	1,357.2	1,213.3	1,332.5

**Table 16.** Fixed-Effect Estimates of Mandarin-English Listeners’ Accuracy in the Mandarin Lexical Decision Task.

Fixed effect	β	*SE*	*z*	*p*
Intercept	−3.297	0.515	−6.401	<.001
Training condition	−0.152	0.834	−0.183	.855
Pronunciation	0.210	0.258	0.814	.416
Training condition: Pronunciation	1.352	1.035	1.306	.192

*SE*: standard error.

Table 17 shows listeners’ responses to the English experimental items, which suggest that these Mandarin–English listeners performed well in the English lexical decision task (in fact, similarly to but not better than the Experiment 2 participants with syllable-initial ambiguous sounds; see Table 10); the ambiguous syllable-final fricative [?] was accepted as an acceptable exemplar of English [f] or [s].

**Table 17.** Percentage of Correct Responses and Response Times (RT), Measured from Target Word Onset, to Experimental Items in the English Lexical Decision Task with Fricatives Occurring in Syllable-Final Position.

Fixed effect	[f]-trained group		[s]-trained group	
	Natural fricatives	Ambiguous fricatives	Natural fricatives	Ambiguous fricatives
*M* % “yes”	87.2	68.1	73.8	71.6
*M* RT “yes” (ms)	1,269.2	1,333.4	1,214.9	1,217.4

We again analyzed the English lexical decision data with *glmer*, and results of the model fit are displayed in Table 18. A significant effect of pronunciation was mediated by an interaction between training condition and pronunciation, which indicated that words containing naturally pronounced fricatives were accepted as existing words more often than words containing ambiguous fricatives for the [f]-trained group only. Overall, these L2 results resembled those of Experiment 2, and again outdid those of Experiment 1.

**Table 18.** Fixed-Effect Estimates of Mandarin–English Listeners’ Accuracy in the English Lexical Decision Task.

Fixed effect	β	*SE*	*z*	*p*
Intercept	−1.578	0.233	−6.783	<.001
Training condition	0.463	0.349	1.327	.184
Pronunciation	0.734	0.174	4.230	<.001
Training condition: Pronunciation	−1.895	0.633	−2.992	.002

*SE*: standard error.

#### 5.3.2 Post-test

**Figure 5.**
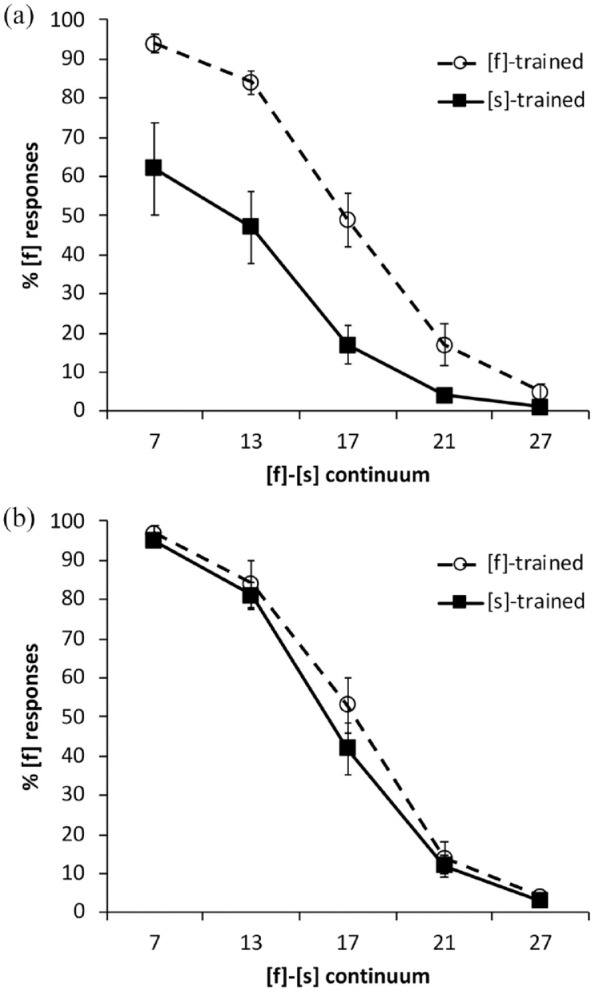
Percentage of [f] responses for the two training groups in Experiment 3 for (a) Mandarin and (b) English. Error bars show standard error of the mean.

The categorization results are shown in Figure 5 for (a) Mandarin and (b) English.

We compared categorization responses between languages and conditions as before with *glmer*. Results of the model fit (Table 19) showed significant effects of training condition and language, and significant interactions between language and continuum step, and language and training condition, again marking an L1–L2 difference in the categorization results. There was also a higher-order three-way interaction. We again checked whether lexical decision scores predicted overall training-consistent categorization responses, and again found null results: *r*(23) = −.073, *p* = .73 for Mandarin, *r*(23) = .370, *p* = .07 for English.

**Table 19.** Fixed-Effect Estimates of Listeners’ Categorization at Post-Test in Both Mandarin and English.

Fixed effect	β	*SE*	*z*	*p*
Intercept	−0.645	0.208	−3.096	.002
Continuum step	0.138	0.159	0.866	.386
Training condition	1.447	0.364	3.974	<.001
Language	1.056	0.269	3.930	<.001
Continuum step: Training condition	−0.069	0.116	−0.590	.555
Continuum step: Language	3.641	0.095	38.378	<.001
Training condition: Language	−2.202	0.660	−3.335	<.001
Continuum step: Training condition: Language	2.067	0.274	7.556	<.001

*SE*: standard error.

In the listeners’ L1, Mandarin (Table 20), the effect of continuum step was significant (i.e., listeners were sensitive to position on the Mandarin [f]-[s] continuum), as was the effect of training condition. In other words, perceptual learning occurred in Mandarin. This is depicted in the separation of the lines in the top panel of Figure 5. For the listeners’ L2, English (Table 21), we only observed a significant effect of continuum step. The effect of training condition was marginally significant, that is, less reliable than the robust effect observed for Mandarin.

**Table 20.** Fixed-Effect Estimates of Mandarin-English Listeners’ Categorization at Post-Test of the Five Steps Selected from the Mandarin [fu]-[su] Continuum.

Fixed effect	β	*SE*	*z*	*p*
Intercept	−1.215	0.316	−3.847	<.001
Continuum step	−1.795	0.166	−10.842	<.001
Training condition	2.141	0.612	3.497	<.001
Continuum step: Training condition	−0.455	0.319	−1.427	.154

*SE*: standard error.

**Table 21.** Fixed-Effect Estimates of Mandarin-English Listeners’ Categorization at Post-Test of the Five Steps Selected from the English [fu]-[su] Continuum.

Fixed effect	β	*SE*	*z*	*p*
Intercept	0.025	0.229	0.110	.912
Continuum step	2.032	0.197	10.330	<.001
Training condition	0.758	0.441	1.715	.086
Continuum step: Training condition	0.478	0.385	1.243	.214

*SE*: standard error.

### 5.4 Discussion

Experiment 3 addressed the same issue as Experiment 2, and the two studies have produced closely similar results. In both cases, Mandarin–English bilingual listeners showed lexically guided perceptual learning in their L1 Mandarin, but not in their L2 English. The materials used in Experiment 3 provided more extensive lexical constraint on the interpretation of the ambiguous sound than had putatively been the case in Experiment 2, but this change did not increase perceptual learning in English. Nor did any effect reveal itself whereby the word-final position of the fricatives in Experiment 3 (an impermissible position in Mandarin) might putatively have led to a pop-out salience, thus influencing lexical processing in English.

## 6 General discussion

Perceptual accommodation to the speech patterns of individual talkers can be language-specific. A listener’s ability—or inability—to adapt rapidly at this level in one language does not necessarily imply that the same will be true of every language in the same listener’s repertoire. This proposal was put forward by Bruggeman and Cutler (2020) on the basis of a study of Dutch emigrants to Australia, who showed adaptation in their (dominant) L2, but not in their (still active) L1. The present results lend support to Bruggeman and Cutler’s proposal regarding language-specificity, and extend it to further patterns beyond positive for L2 and negative for L1; here our Mandarin-native groups displayed the reverse pattern (clearer learning effects were observed in L1 than in L2), while our English-native group showed learning in both L1 and L2.

Note that even though the sample sizes were smaller than we had hoped to have been able to achieve, this pattern of results was reliable. The availability of this type of perceptual accommodation is not simply an advantage enjoyed by one language—the first-learned, the more dominant, or whatever one might propose—over others. It can vary, with any pattern being possible across any set of familiar languages.

However, it is quite puzzling why such variation should ever occur, given how useful this kind of accommodation can be. The speed with which listeners in general adjust to talkers whom they have never before heard is surely one of the major pillars of the robustness of speech perception. Such use of external knowledge (in this case from the lexicon) to tailor phoneme recognition in a talker-specific manner has been established across languages of Europe, the United States, and Asia (Chan et al., 2020; Clarke-Davidson et al., 2008; Mitterer et al., 2010; Norris et al., 2003) and for listeners across an age range from childhood (McQueen et al., 2012) to “senior citizen” (Scharenborg & Janse, 2013). It has been observed in listeners’ L1 and in their L2 (although only Bruggeman & Cutler, 2020 and our own work, Burchfield et al., 2023) could reveal language-specific variation, by testing in two languages with a single listener group). These two cases of adaptation absence seem to share no obvious feature offering a solution to this puzzle.

Such a feature could in principle be sought in experimental factors, or in differences between the participant populations. Our perceptual learning task relies on phonetic categorization, in a long speech science tradition driven more by practical than by theoretical considerations. Speech perception accuracy requires listeners to perceive distinctions (*bad* is not *pad* or *bed* or *bat* or indeed *pet*) but it does not actually require categorization; speech perception research in fact provides no empirical evidence for a processing level at which *bad* is deemed to consist of three sounds, which need to be separately considered, labeled, or recognized as independent entities. In the early days of psycholinguistics, there was extensive concern about identification of “units of perception” (i.e., the form in which phonological structure is recorded in the lexicon, and in consequence the target of phonological processing of speech input by listeners). No accepted theory resulted from this debate, however, and the eventual accumulation of empirical evidence of multiple activation of lexical candidates, and active competition between them, led to spoken-word recognition models (Luce & Pisoni, 1998; McClelland & Elman, 1986; Norris, 1994) based on this process. Although cross-word similarities were expressed in terms of sublexical perceptual units such as phonemes, the models’ function was centered on the entities stored in the lexicon. So far, the approach taken in these models has stood the test of time.

Despite this clear theoretical agreement, most of the relevant empirical evidence on which it is based has been collected using tasks in which listeners are required to make judgments about such sublexical units, and the phoneme in particular. Thus phonetic categorization (e.g., Peterson & Barney, 1952, for vowels; Miller & Nicely, 1955, for consonants) requires judgment of whether a sound is more like one phoneme than others; phoneme-monitoring (e.g., Foss, 1969) requires detection of prespecified phonemes (e.g., a word beginning with /b/); word reconstruction (e.g., Van Ooijen, 1996) requires lexical recognition achieved by altering a single phoneme in the heard input. Notably, following instructions to respond at the appropriate level seems to create no significant problems at all for participants in such studies. It has been argued that the ease with which phonemic apprehension is achievable, despite it not being a necessary component of speech perception, arises in our experience of alphabetic literacy (see, for example, Morais, 2021, in particular the account of an illiterate poet with superlative rhyming skills unaccompanied by phonemic awareness).

The first option of experimental factors influencing our result pattern does not involve any task-specific feature that would set perceptual learning apart from any other task requiring such abstraction from the speech signal. In our particular study, then, could the separate materials sets be driving the results patterns? That does not seem likely, given that the Mandarin materials always produced a positive learning result, the initial set of English materials (Experiments 1 and 2) did the same with English-native listeners at least, and the Experiment 3 English materials had elicited a positive result in prior work. Not even the overall strength of the exposure training was important: the lowest L2 lexical decision scores in the present study were in Experiment 1, where they accompanied a positive L2 learning effect.

The location of the experimental investigation also does not appear to be crucial. As expected from results showing language immersion to be an irrelevant factor (Drozdova et al., 2016; Schuhmann, 2014), this was also no issue here; Sydney is an English-speaking environment (but Mandarin as well as English gave a positive learning result); Hong Kong is a multilingual site (but Mandarin produced the clearest positive finding).

Another experimental factor, language phonology, was raised earlier: prior cross-language comparisons involved only the typologically alike English, Dutch, and German. But again, such similarity does not appear to be driving the results here. Mandarin and English are from quite different language families; although the present experiments involved the same consonant contrast as had produced robust learning in other languages, the materials were typologically dissimilar and varied on several phonological dimensions. Mandarin has fewer vowels and fewer consonants than English, and it has simpler syllable structure and uses no affixal morphology. In the suprasegmental domain, however, Mandarin uses lexical tones to distinguish word meaning, with every syllable bearing a tone and every tone being realizable on every vowel. It is thus unclear which phonology counts as the more complex. In any case, such differences are no bar to perceptual learning, as shown by the results of our Experiment 1 as well as by the finding of Chan et al. (2020) in an experiment with English-dominant participants with heritage knowledge of Cantonese (that differs from English in much the same ways as Mandarin does). Syllable structure had the opportunity to reveal itself as a factor in our study in that the ambiguous sounds in the English materials in Experiment 3, occurring in the position of maximal lexical information at the end of the word, were word- and syllable-final and, unlike the syllable-initial ambiguous sounds in the English stimuli of Experiments 1 and 2, illegally positioned for fricatives in Mandarin. Nonetheless the results across the experiments were comparable and no difference between Experiments 2 and 3 seems attributable to relative complexity.

The structure of phoneme inventories can also influence phoneme perception. For instance, the consonant:vowel ratio affects listeners’ expectations of coarticulatory effects in speech (thus Spanish listeners, whose language has many more consonants than vowels, expect more variability in vowels, while Dutch listeners, with a balanced inventory, expect equivalent vowel and consonant variation; Costa et al., 1998). Likewise, phoneme similarity in inventory subsets affects phonemic realization: a phoneme with few close neighbors is more variable in how it is realized, while a phoneme with many close competitors has more constant realizations (see Bradlow, 1995; Flege, 1989 for English vs. Spanish vowels, using categories appearing in both inventories). Relative variability in turn influences listeners’ attention to articulatory detail in identifying phonemes (Wagner, 2013); the more exact the realization needs to be in L1, the more attention listeners give it.

If, in perceptual learning, such attentional requirements differ across the phonemic endpoints involved, the relative adaptation in each direction could be affected (recall that Drozdova et al., 2016, observed such an adaptation asymmetry with their the lateral/rhotic distinction). In the present study that would cause a shift of judgments across the continuum for one group but not for the other. Here, we indeed recorded some tendency for our /s/-trained groups to show slightly greater continuum shifts (in comparison to the relevant pretests) than the /f/-trained groups; so might this reflect the more populated sibilant space of Mandarin? This also seemed to us an unlikely conclusion given that the pattern was shown by both English-native and Mandarin-native listeners, and with both languages (besides being statistically insignificant).

Turning then to the second option, could it be that the participant populations themselves determine the effects? Bruggeman and Cutler suggested that the asymmetric pattern they observed might reflect the relative number of interlocutors with whom their participants conversed in their current everyday life. Since perceptual learning has been observed even in childhood, as well as in many languages in many countries, it is probably fair to assume that those participants had once been able to adapt to unfamiliar talkers in their L1. The observed lack of L1 effect might then spring from the fact that although they conversed regularly in L1, it was usually with their family, hence with a restricted set of familiar talkers for whom, effectively, no adaptation was required. (This could in turn imply that their ability to adapt in this way in L1 was just in temporary suspension, and potentially would be recovered if they spent significant time in an L1 environment once more.) A similar argument was proposed for the lack of effect in a heritage language, reported by Cutler et al. (2019).

Although this explanation is attractive in the sense that it is a manipulable factor offering not only good news for emigrants but also a potential route to L2 listening improvement for learners (go and talk to more people!), it is hard to see it as an explanation of the present asymmetric findings. The English-native learners of Mandarin who showed perceptual learning in Mandarin are the least likely of our participant groups to enjoy a large number of interlocutors in that language, whereas the Mandarin-native participants of Experiments 2 and 3 are likely to be in the opposite situation—with their university classes and the many media opportunities around them, even including public transport announcements in English and a large tourist presence, they cannot be said to be hearing English only from a few familiar talkers. Such a suggestion cannot be definitively ruled out, but some additional factors may be needed in the present case.

It seems unlikely that age is a factor; Bruggeman and Cutler’s participants were older than the average experimental participant, but the present participants were not. Likewise, linguistic proficiency did not cause the variation observed in either study. All participants here were competent in both languages, and better or worse relative performance in the exposure task never predicted presence versus absence of perceptual learning. Bruggeman and Cutler’s emigrants who showed no adaptation in their L1 nonetheless reported regular use of the L1, and achieved accordingly high scores in a proficiency test; the present group of listeners who showed no perceptual learning in their L2 used that language regularly when listening to lectures, and they too showed high proficiency scores.

One effect that has been well studied in the L2 learning literature, but without its implications for language perception as yet having been fully assessed, is the so-called “fossilization” that leads competent L2 users to stop improving once they have reached a level at which they can use their L2 effectively, albeit far from perfectly (Birdsong, 1992, 1999). We consider that this state may well hold for our Experiment 2 and 3 listeners, but is less likely to be true of our Experiment 1 listeners, many of whom were currently engaged in Mandarin classes; thus it may be relevant to the absent adaptation in L2 in Experiments 2 and 3, compared with the successful adaptation in L2 in Experiment 1. (Bruggeman and Cutler’s participants on the contrary were dominant in L2; the language in which no adaptation appeared was their L1.) If such L2 patterns are indeed of relevance in speech perception, this could not only shed light on the present pattern of results, but it could also suggest new ways in which to view, and potentially train, L2 users’ adaptation to talker variation.

Thus, we cannot as yet definitively identify the precise triggers that lead lexically guided perceptual learning to fail, nor exactly where such a failure will occur (in L1 in Bruggeman and Cutler’s study, and in one of the L2 cases, but not the other, in the present study). But it is fully clear that this form of adaptation to talkers is language-specific, and any language user may experience an asymmetry between its availability in one and another language. The factors that influence the appearance of perceptual learning in a given language are potentially many, and a rich field of new investigation may open up for this research topic.

## Appendix A

### Lexical decision materials

**Table:** Mandarin Items Containing Fricatives as First Phoneme of Second Syllable Used in All Perceptual Learning Experiments.

[f] words	[s] words
经费 jing1 fei4(n.) funds	场所 chong3 suo3(n.) place
咖啡 ka1 fei1(n.) coffee	无私 wu2 si1(adj.) selfless
处分 chu3 fen4(v.) punish	伴随 ban4 sui2(v.) accompany
惩罚 cheng2 fa2(v.) penalize	灰色 hui1 se4(n.) gray
无非 wu2 fei1(adv.) nothing but	压缩 ya1 suo1(v.) compress
每逢 mei3 feng2(adv.) every time	跟随 gen1 sui2(v.) follow behind
勤奋 qin2 fen4(adj.) diligent	运送 yun4 song4(v.) transport
气愤 qi4 fen4(adj.) furious	歌颂 ge1 song4(v.) praise
面粉 mian4 fen3(n.) flour	打扫 da3 sao3(v.) sweep
嘲讽 chao2 feng3(v.) ridicule	寓所 yu4 suo3(n.) residence
供奉 gong4 feng4(v.) worship	推算 tui1 suan4(v.) reckon
裂缝 lie4 feng4(n.) crack	钢丝 gang1 si1(n.) wire
奶粉 nai3 fen3(n.) milk powder	苦思 ku3 si1(v.) think hard
颓废 tui2 fei4(adj.) decadent	貌似 mao4 si4(adv.) seemingly
直飞 zhi2 fei1(v.) have direct flight	半死 ban4 si3(adj.) almost dead
王妃 wang2 fei1(n.) princess	寒酸 han2 suan1(adj.) poor
绑匪 bang3 fei3(n.) kidnapper	教唆 jiao4 suo1(v.) instigate
停飞 ting2 fei1(v.) stop flying	猥琐 wei3 suo3(adj.) wretched
劫匪 jie2 fei3(n.) robber	淤塞 yu1 se4(v.) block up
门扉 men2 fei1(n.) door leaf	暖色 nuan3 se4(n.) warm color

**Table:** English fricative-medial items used in Experiments 1 and 2.

[f]-medial words	[s]-medial words
affair	accent
afraid	answer
before	asleep
bonfire	assert
coffin	basic
comfort	basin
confer	beside
conflict	cassette
dreadful	classic
effect	consult
painful	counsel
inform	crusade
pamphlet	despair
perform	gangster
prefer	insane
profit	inside
refer	instead
reflect	insult
reform	listen
unfair	restore

**Table:** English fricative-final items used in Experiment 3.

[f]-final words	[s]-final words
aloof	across
autograph	awareness
beef	bias
behalf	bliss
belief	crease
bulletproof	dress
dandruff	embrace
debrief	entice
dwarf	eyewitness
earmuff	harrass
enough	hideous
handcuff	immerse
laugh	necklace
loaf	noose
midwife	pierce
plaintiff	promise
proof	remorse
rough	replace
whooping-cough	twice
wildlife	unless

## Appendix B

### English proficiency admission requirements at CUHK

Students must have (a) obtained a degree from a university in Hong Kong or taken a degree program of which the medium of instruction was English; or (b) achieved scores in the following English Language tests as indicated: TOEFL: 550 (Paper-based)/79 (internet-based); IELTS (Academic): 6.5; GMAT (Verbal): Band 21; or (c) obtained a pass grade in English in one of the following examinations: Hong Kong Advanced Level Examination (AS Level); Hong Kong Higher Level Examination; CUHK Matriculation Examination; General Certificate of Education Examination (GCE) Advanced Level (A-Level)/Advanced Subsidiary Level (AS Level); or (d) achieved Level 4 or above in the English Language subject of the Hong Kong Diploma of Secondary Education (HKDSE) Examination; or (e) obtained a recognized professional qualification, with the examination conducted in English.
